# Propofol inhibits proliferation, migration, and invasion but promotes apoptosis by regulation of Sox4 in endometrial cancer cells

**DOI:** 10.1590/1414-431X20176803

**Published:** 2018-02-26

**Authors:** Qing Du, Jia Liu, Xuezhi Zhang, Xin Zhang, He Zhu, Ming Wei, Shilei Wang

**Affiliations:** 1 Department of Anesthesiology, The Affiliated Hospital of Qingdao University, Qingdao, China; 2 Department of Emergency, The Affiliated Hospital of Qingdao University, Qingdao, China

**Keywords:** Endometrial cancer, Propofol, Cell proliferation, Apoptosis, Sox4, Wnt/β-catenin

## Abstract

Propofol is an intravenous sedative hypnotic agent of which the growth-inhibitory effect has been reported on various cancers. However, the roles of propofol in endometrial cancer (EC) remain unclear. This study aimed to explore the effects of propofol on EC *in vitro* and *in vivo*. Different concentrations of propofol were used to treat Ishikawa cells. Colony number, cell viability, cell cycle, apoptosis, migration, and invasion were analyzed by colony formation, MTT, flow cytometry, and Transwell assays. In addition, the pcDNA3.1-Sox4 and Sox4 siRNA plasmids were transfected into Ishikawa cells to explore the relationship between propofol and Sox4 in EC cell proliferation. Tumor weight *in vivo* was measured by xenograft tumor model assay. Protein levels of cell cycle-related factors, apoptosis-related factors, matrix metalloproteinases 9 (MMP9), matrix metalloproteinases 2 (MMP2) and Wnt/β-catenin pathway were examined by western blot. Results showed that propofol significantly decreased colony numbers, inhibited cell viability, migration, and invasion but promoted apoptosis in a dose-dependent manner in Ishikawa cells. Moreover, propofol reduced the expression of Sox4 in a dose-dependent manner. Additionally, propofol significantly suppressed the proportions of Ki67^+^ cells, but Sox4 overexpression reversed the results. Furthermore, *in vivo* assay results showed that propofol inhibited tumor growth; however, the inhibitory effect was abolished by Sox4 overexpression. Moreover, propofol inhibited Sox4 expression via inactivation of Wnt/β-catenin signal pathway. Our study demonstrated that propofol inhibited cell proliferation, migration, and invasion but promoted apoptosis by regulation of Sox4 in EC cells. These findings might indicate a novel treatment strategy for EC.

## Introduction

Endometrial cancer (EC) is one of the most common malignancies of the female reproductive system (1,2). Globally, more than 200,000 women suffer from EC every year, and the mortality rate is just behind ovarian cancer and cervical cancer (3). To date, the primary treatment for EC is surgery followed by adjuvant radiation therapy and chemotherapy (4). However, these therapies have not effectively reduced the risk of EC mortality. The recurrence rates of EC are higher than 60-80% two to three years after surgery (5). Currently, an increasing number of studies have demonstrated that anesthetics and anesthetic techniques have beneficial effects on postoperative recurrence and could improve survival rate in various cancers (6,7). However, the effect of anesthetics on EC and its molecular mechanism remain unclear.

Propofol is a common intravenous anesthetic that can induce and restore general anesthesia rapidly (8). Recently, propofol has been widely reported to play an anti-tumor role in various cancers (9). Yang et al. (10) reported that propofol could inhibit lung cancer cell viability and promote apoptosis. Moreover, several studies demonstrated that propofol induced cervical cancer cell apoptosis, inhibited pancreatic cancer progression, inhibited ovarian cancer cell invasion, and suppressed breast cancer cell proliferation and migration (11–14). However, little information is available about the anti-tumor effect of propofol on EC.

Sex-determining region Y-box 4 (Sox4) is a member of the sex-determining region Y (SRY)-related high-mobility-group (HMG-box) family (15). Accumulating evidence has demonstrated that Sox4 is a key regulator in multiple cellular processes and down-regulation of SOX4 could inhibit cancer cells metastasis (16,17). However, whether Sox4 was involved in regulation of cell growth and metastasis in EC cells remains not fully investigated. Therefore, in our study, we aimed to explore the effects of propofol on cell proliferation, migration, invasion and apoptosis of EC cells, and uncover the relationship between propofol and Sox4 in EC cells. Furthermore, the relevant signal pathway was examined to reveal the potential molecular mechanisms. Our study might provide a new idea and theoretical basis for treatment of EC.

## Material and Methods

### Cell culture

The EC cell line of Ishikawa was obtained from JCRB Cell Bank (Japan). Briefly, Ishikawa cells were grown in Dulbecco’s modified Eagle medium (DMEM)/F12 (Sigma-Aldrich, USA) supplemented with 10% fetal bovine serum (FBS, Gibco Life Technologies, USA) at 37°C, 5% CO_2_ and 95% humidity atmosphere (18). Propofol purchased from Sigma-Aldrich was diluted in dimethyl sulfoxide (DMSO, Sigma-Aldrich) for *in vitro* and *in vivo* assays (19). These cells were cultured for 24 h, followed by treatment with the different concentrations of propofol (2, 4, and 6 μg/mL). The cells used as the control group were cultured in 0.1% DMSO for 24 h.

Animal health and protocols were in accordance with the guidelines of the Institutional Animal Care and Use Committee of The Affiliated Hospital of Qingdao University.

### Plasmids and siRNA transfection

The Sox4 and β-catenin small interference RNA (siRNA) were constructed by GenPharma (China) to inhibit the Sox4 and β-catenin expressions. The sequence of si-Sox4 is 5′-UUU GCC CAG CCG CUU GGA GAU CUC G-3′; si-NC sequence is 5′-UUC UCC GAA CGU GUC ACG-3′; si-β-catenin sequence is 5′-CAC CTC CCA AGT CCT TTA T-3′, its control sequence is 5′-TTC TCC GAA CGT GTC ACG T-3′. The pcDNA3.1-Sox4 or pcDNA3.1-β-catenin plasmids were also structured by GenPharma (Shanghai) to overexpress the Sox4 and β-catenin expressions. All transfection cells were accomplished using Lipofectamine 2000 (Invitrogen, USA), according to the manufacturer’s instructions. After transfection for 48 h, the supernatants were collected for subsequent analyses.

### Colony formation assays

For colony formation assay, about five hundred cells were added to a 6-well culture plate and transfected for 48 h. Then, the cells were cultured for 14 d at 37°C. After this, cells were stained with 10% Giemsa (Merck, Germany) for 30 min. Colonies containing ≥50 cells were counted under a microscope (Olympus, Japan). Each experiment was repeated three times.

### Cell viability

Cell viability was determined by using 3-(4, 5-dimethylthiazol-2-yl)-2 5-diphenyl-2Htetrazolium bromide (MTT) colorimetric assay according to standard methods described before (20). In brief, Ishikawa cells (5 × 10^3^ cells per well) were seeded in 96-well plates and incubated for 24 h at 37°C. Then, 10 μl MTT (0.5 mg/mL, Sigma-Aldrich) was supplemented into each well and incubated at 37°C, in 5% CO_2_ for another 3 h. Afterwards, 100 mL DMSO (Sigma-Aldrich) was added to dissolve the formazan crystals. The absorbance was examined at 450 nm using a microplate reader (Bio-Rad, Hercules, USA).

### Cell cycle analysis

Cell cycle was detected by flow cytometry assay. In brief, following treatment with 4 μg/mL of propofol for 24 h, Ishikawa cells (10^5^) were harvested and washed twice with ice-cold phosphate-buffered saline (PBS). These cells were suspended gently in 70% chilled ethanol at 4°C overnight. Then cells were re-suspended in 500 μL of PBS containing 0.2 mg/mL RNaseA and 50 μg/mL PI and were incubated for 30 min at room temperature in the dark. The proportion of Ishikawa cells in G0/G1, S and G2/M phases were determined by the ModFit software (Verity Software House, USA) (21).

### Apoptosis assay

Cell apoptosis was detected by using Annexin V-FITC/PI apoptosis detection kit (Beijing Biosea Biotechnology, China). In brief, cells (10^5^ cells/well) were seeded in 6 well-plates. Treated cells were washed twice with cold PBS and re-suspended in buffer followed by addition of 5 μl Annexin V-FITC and 10 μL PI. After incubation for 1 h at room temperature in the dark, the adherent and floating cells were measured with flow cytometer (Beckman Coulter, USA) using FlowJo software (Tree Star, Inc., USA) to differentiate apoptotic cells.

### Cell migration and invasion assay

Migration of Ishikawa cells was investigated by using a 24-well Transwell chemotaxis chamber (Costar, USA) with an 8 μm pore size membrane. In brief, Ishikawa cells were suspended in 200 µL serum-free DMEM medium, and these cells were filled in the upper chamber. Then, 600 µL complete medium was added to the lower compartment and incubated for 12 h. Then the non-migrated cells were removed from the upper surface with a cotton swab, and the migrated cells on the lower side of the insert were fixed and stained with hematoxylin for 15 min and counted under a microscope (Olympus). For cell invasion, the experimental methods are similar to cell migration except the inserts were coated with BD Matrigel^TM^ Matrix (BD Biosciences, USA) (22).

### Immunohistochemistry analysis of Ki67 positive cells

After treatment of 4 μg/mL of propofol for 24 h, cells were washed with PBS and fixed in chilled methanol-acetone (1:1) at -20°C for 10 min. After this, cells were washed again with PBS, and blocked with 3% BSA for 1 h in humidified CO_2_. Then, these cells were incubated with an anti-Ki67 antibody (ab16667, Abcam, UK) for 20 h at 4°C with intermittent shaking. Subsequently, cells were washed with sterile PBS and incubated with an anti-rabbit antibody (ab205718, Abcam) conjugated with Fluorescein isothiocyanate (FITC) for 4 h at 37°C. Finally, cells were stained with 3′-doaminobenzidine tetrahydrochloride (DAB) for 10 min and observed under a Nikon Eclipse 80i fluorescence microscope (Nikon, Japan). The percentages of the positive stained cells were calculated.

### Evaluation of tumorigenicity

The tumorigenicity was analyzed by subcutaneously injecting 2 × 10^6^ cells into the flanks of 6-week-old female BALB/c-nu mice. Animal health and protocols were in accordance with the guidelines of the Institutional Animal Care and Use Committee of The Affiliated Hospital of Qingdao University. In brief, these mice were divided randomly into three groups. Treatment and transfected cells were plated on 6-well plates until cells were 70-80% confluent. Then, each group was injected subcutaneously into both sides of the flank region with 0.2 mL of 10^9^ cells suspension. After four weeks, the tumor weight was measured according to a previous study (23). All studies involving animals were approved by the Ethics Committee of The Affiliated Hospital of Qingdao University.

### Quantitative real time PCR (qRT-PCR) analysis

Total RNA was extracted using TRIzol reagent (Invitrogen), and was reverse-transcribed into complementary DNAs (cDNAs) using the PrimeScript RT reagent kit (Takara, Japan) according to the manufacturer’s instructions. qRT-PCR was performed using Takara SYBR Premix Ex Taq II (Takara). GAPDH was used for normalization. Primers used for mRNA expression were obtained from the PrimerBank database (http://pga.mgh.harvard.edu/primerbank/). The data were analyzed with the 2^-ΔΔCt^ method. PCR primers are as follows: Sox4 forward, 5′-GTG AGC GAG ATG ATC TCG GG-3′; Sox4 reverse, 5′-CAG GTT GGA GAT GCT GGA CT C-3′. GAPDH forward, 5′-CAT CAC CAT CTT CCA GGA GCG-3′; GAPDH reverse, 5′- TGA CCT TGC CCA CAG CCT TG-3′.

### Western blot

The protein in Ishikawa cells used for western blot was extracted using RIPA lysis buffer (Beyotime Biotechnology, China) supplemented with protease inhibitors (Roche, Switzerland). The proteins were separated by sodium dodecyl sulfate polyacrylamide gel electrophoresis (SDS-PAGE). After rinsing, the proteins were transferred onto polyvinylidene fluoride (PVDF) membranes and blocked with 5% skim milk, and blotted with the appropriate primary antibodies of P21 (ab219811), Cyclin E1 (ab52189), Cyclin D1 (ab61758), Bid (ab63541), BCL-2-Associated X (Bax, ab32503), active caspase 3 (ab49822), MMP9 (ab38898), MMP2 (ab37150), Sox4 (ab86809), β-catenin (ab6302) GAPDH (ab8245, Abcam, UK) (all dilutions 1:1000) and secondary antibodies. The chemiluminescence assay (Millipore, Germany) was used to detect the antigen-antibody complexes (24). The relative density was examined using Image Lab™ Software (Bio-Rad, China).

### Statistical analysis

All experiments were repeated three times. The results of multiple experiments are reported as means±SD. Statistical analyses were performed using SPSS 19.0 statistical software (SPSS Inc., USA). The P-values were calculated using one-way analysis of variance (ANOVA). A P-value <0.05 was considered to indicate a statistically significant result.

## Results

### Propofol inhibited cell proliferation in Ishikawa cells

**Figure 1.**
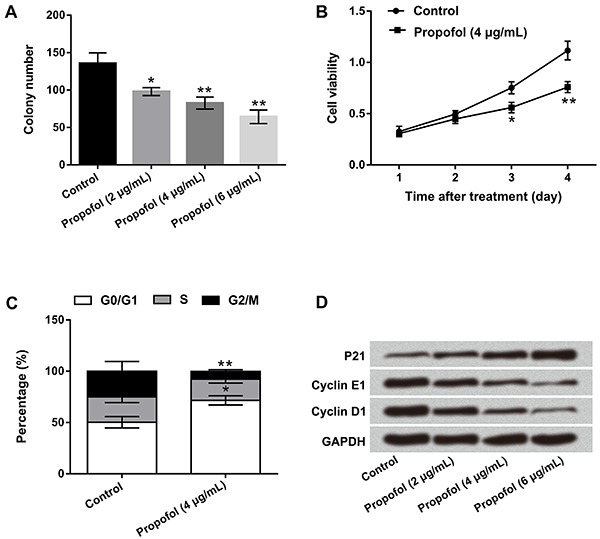
Propofol inhibited cell proliferation in Ishikawa cells. Human EC Ishikawa cell line was exposed to different concentrations of propofol (2, 4, and 6 μg/mL). After treatment for 24 h (*A*) the colony number (*B*) cell viability (*C*) cell cycle and (*D*) the protein levels of cell cycle-related factors (P21, Cyclin E1 and Cyclin D1) were detected by colony formation, MTT, flow cytometry, and western blot assays. EC: endometrial cancer; MTT: 3-(4,5-dimethylthiazol-2-yl)-2 5-diphenyl-2Htetrazolium bromide. Data are reported as means±SD. *P<0.05; **P<0.01, compared to control (ANOVA).

Colony formation assay results showed that propofol significantly decreased the colony number in a dose-dependent manner compared to the control group (P<0.05 or P<0.01, Figure 1A). Additionally, we used 4 μg/mL of propofol to examine cell viability. MTT assay results showed a significant decreased in the propofol treatment group compared to the control group and significant differences were found at 3 and 4 days (P<0.05 or P<0.01, Figure 1B). In terms of cell cycle analysis, propofol remarkably increased the proportion of cells in G0/G1 phase (P<0.05, Figure 1C). Additionally, western blot results showed that propofol up-regulated P21 expression, but down-regulated Cyclin E1 and Cyclin D1 expressions in a dose-dependent manner (Figure 1D). All data indicated that propofol inhibited cell proliferation in Ishikawa cells.

### Propofol promoted cell apoptosis in Ishikawa cells

**Figure 2.**
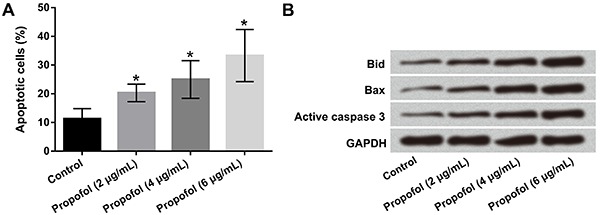
Propofol promoted cell apoptosis in Ishikawa cells. Human EC Ishikawa cell line was exposed to different concentrations of propofol (2, 4, and 6 μg/mL). After treatment for 24 h (*A*) the percentage of apoptotic cells and (*B*) the protein levels of Bid, Bax and active caspase 3 were examined by flow cytometry and western blot. EC: endometrial cancer; Bax: B-Cell Lymphoma-2-Associated X. Data are reported as means±SD. *P<0.05, compared to control (ANOVA).

As shown in Figure 2A, propofol enhanced the percentage of apoptotic cells in a dose-dependent manner in Ishikawa cells compared to the control group (P<0.05). In addition, the expression levels of Bid, Bax and activate caspase 3 were significantly increased by propofol at 4 μg/mL and 6 μg/mL (Figure 2B). The results showed that propofol promoted cell apoptosis in Ishikawa cells.

### Propofol inhibited cell migration and invasion in Ishikawa cells

**Figure 3.**
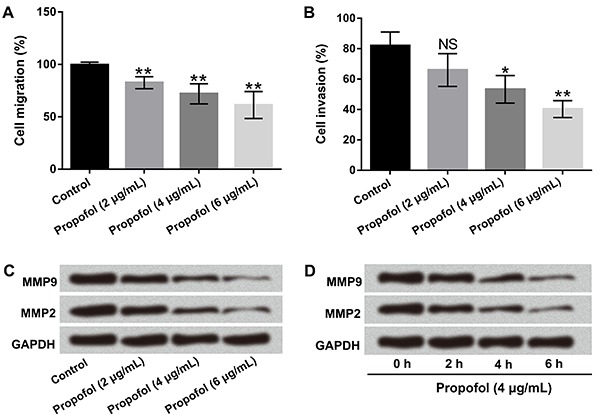
Propofol inhibited cell migration and invasion in Ishikawa cells. Human EC Ishikawa cell line was exposed to different concentrations of propofol (2, 4, and 6 μg/mL). After treatment for 24 h, (*A*) cell migration, (*B*) cell invasion, (*C*) and protein levels of MMP9 and MMP2 were determined by transwell assay and western blot. Then, cells were treated with 4 μg/mL of propofol and (*D*) the protein levels of MMP9 and MMP2 at different time points (0, 2, 4, and 6 h) were determined by western blot. EC: endometrial cancer; MMP9: matrix metallopeptidase 9; MMP2: matrix metalloproteinase-2. Data are reported as means±SD. *P<0.05; **P<0.01, compared to control (ANOVA). NS: not significant.

Results showed that propofol inhibited cell migration at 2, 4, and 6 μg/mL compared to the control group (Figure 3A, P<0.01). Propofol also decreased cell invasion significantly at 4 μg/mL and 6 μg/mL (P<0.05, P<0.01, Figure 3B). Moreover, the expression levels of MMP9 and MMP2 at different concentrations of propofol (2, 4, and 6 μg/mL) were examined by western blot. Results showed that propofol remarkably down-regulated MMP9 and MMP2 expressions in a dose-dependent manner (Figure 3C). Furthermore, we selected 4 μg/mL of propofol and analyzed the expression levels of MMP9 and MMP2 at different times (2, 4, and 6 h). Results showed that propofol also significantly down-regulated these two factors in a time-dependent manner (Figure 3D). The above results indicated that propofol inhibited cell migration and invasion in Ishikawa cells.

### Propofol inhibited cell proliferation by regulation of Sox4

**Figure 4.**
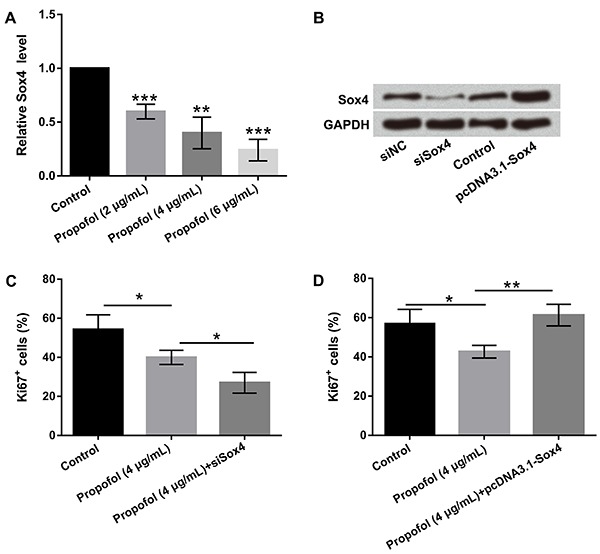
Propofol inhibited cell proliferation by regulation of Sox4 in Ishikawa cells. Human EC Ishikawa cell line was exposed to different concentrations of propofol (2, 4, and 6 μg/mL) for 24 h and (*A*) the mRNA expression of Sox4 was detected by qRT-PCR. Then, pcDNA3.1-Sox4 and Sox4 siRNA were transfected into Ishikawa cells and (*B*) the protein level of Sox4 in overexpression and silencing of Sox4 were measured by and western blot. In *C* and *D*, the percentage of Ki67^+^ cells was analyzed by immunohistochemistry analysis. EC: endometrial cancer; Sox4: sex-determining region Y-box 4; siRNA: small interference RNA; qRT-PCR: quantitative real time PCR. Data are reported as means±SD. *P<0.05; **P<0.01; ***P<0.001 (ANOVA).

To confirm the relationship between propofol and Sox4 on cell proliferation in EC, pcDNA3.1-Sox4 and Sox4 siRNA and corresponding controls were transfected into Ishikawa cells. As shown in Figure 4A, the expression of Sox4 was significantly down-regulated by propofol in a dose-dependent manner compared to the control group (P<0.01 or P<0.001). In addition, the protein level of Sox4 was decreased by silencing of Sox4 as well as increased by overexpression of Sox4, indicating that the transfection of Soxe4 was successful and could be used in subsequent experiments (Figure 4B). As shown in Figure 4C, propofol decreased the percentage of Ki67^+^ cells, while propofol together with Sox4 silencing further decreased the percentage of Ki67^+^ cells compared to the propofol group (P<0.05). However, the inhibitory effects of propofol on Ki67^+^ cells were significantly abolished by Sox4 overexpression (P<0.05 or P<0.01, Figure 4D). These results suggested that Sox4 overexpression could alleviate the inhibitory effect of propofol on cell proliferation by down-regulation of Sox4 in Ishikawa cells.

### Propofol inhibited tumor formation by regulation of Sox4 *in vivo*

**Figure 5.**
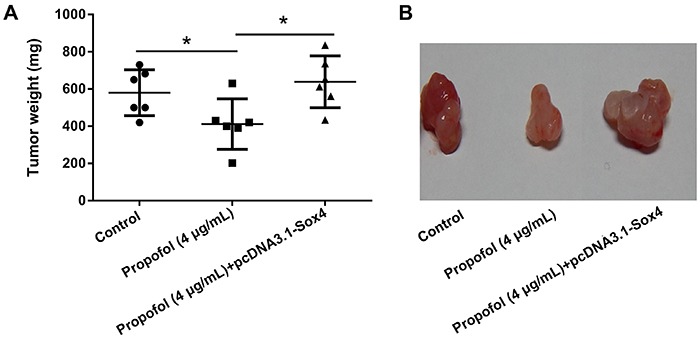
Propofol inhibited tumor formation via regulation of Sox4 *in vivo*. Human EC Ishikawa cell line was exposed to 4 μg/mL of propofol, and pcDNA3.1-Sox4 was transfected into Ishikawa cells to overexpress Sox4 expression. *A*, Tumor weight was detected by xenograft tumor model assay. *B*, Images of tumors obtained from the mice in different treatment groups. EC: endometrial cancer; Sox4: sex-determining region Y-box 4. *P<0.05 (ANOVA).

To further clarify the effect of propofol and Sox4 on EC, xenograft tumor model assay was used to examine the tumor formation *in vivo*. Results in Figure 5A and 5B showed that propofol remarkably decreased tumor weight compared to the control group (P<0.05), but propofol together with Sox4 overexpression significantly increased tumor weight compared to the propofol group (P<0.05). These data indicated that propofol inhibited tumor formation by regulation of Sox4 *in vivo*.

### Propofol inhibited Sox4 expression via inactivation of Wnt/**β**-catenin signal pathway

**Figure 6.**
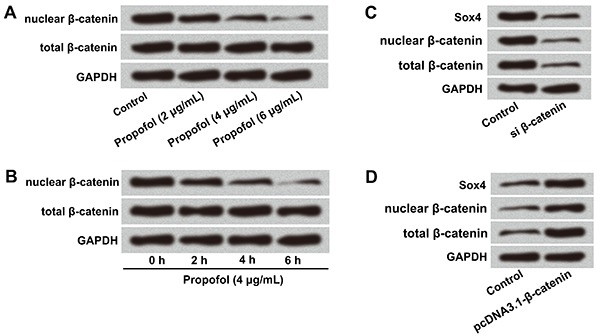
Propofol inhibited Sox4 expression via inactivation of Wnt/β-catenin signal pathway. Human EC Ishikawa cell line was exposed to different concentrations of propofol (2, 4, and 6 μg/mL). The expressions of nuclear β-catenin and total β-catenin (*A*) in different concentration of propofol and (*B*) in different time points (0, 2, 4, and 6 h) were examined by western blot. The β-catenin siRNA and pcDNA3.1-β-catenin were transfected into Ishikawa cells. In *C* and *D*, the expressions of Sox4, nuclear β-catenin and total β-catenin in overexpressing and silencing β-catenin transfected cells were also detected by western blot. EC: endometrial cancer; Sox4: sex-determining region Y-box 4; siRNA: small interference RNA.

Western blot results showed that propofol remarkably reduced nuclear β-catenin expression in a dose-dependent manner and time-dependent manner (Figure 6A and 6B). There was no difference in total β-catenin expression. In addition, silencing of β-catenin down-regulated Sox4, nuclear β-catenin and total β-catenin expressions, while overexpression of β-catenin up-regulated the expressions of these factors (Figure 6C and 6D). These data indicated that propofol inhibited Sox4 expression via inactivation of Wnt/β-catenin signal pathway.

## Discussion

In our study, we explored the effects of propofol on EC *in vitro* and *in vivo*. We found that propofol significantly decreased cell proliferation, migration, and invasion but promoted apoptosis in a dose-dependent manner in Ishikawa cells. Moreover, propofol could reduce Sox4 expression, and overexpression of Sox4 abolished the inhibitory effect of propofol on cell proliferation. Furthermore, the *in vivo* experiment revealed that propofol inhibited tumor weight via regulation of Sox4. As to the mechanism, we found that propofol inhibited Sox4 expression via inactivation of Wnt/β-catenin signal pathway.

Anesthetics are unavoidable for cancer patients who receive surgical treatment (25). Several studies have reported that anesthetics could influence cell growth and metastasis and contribute to enhance survival rate of patients who have accepted cancer surgery in a variety of cancers (22,26). Propofol is one of these anesthetics and has been extensively researched. In *in vitro* experiments, Zhang et al. showed that propofol induced cell proliferation and invasion in gallbladder cancer cells through activation of Nrf2 (27). Contrarily, Liu et al. demonstrated that propofol inhibited cell growth and invasion of pancreatic cancer cells through regulation of the miR-21/Slug signaling pathway (28). However, to the best of our knowledge, the effect of propofol on EC has not been reported in existing studies. In our study, we found that propofol exerted anti-tumor effect on EC by inhibiting cell proliferation, metastasis and promoting apoptosis.

Sox4 is a regulatory factor involved in tumorigenesis and tumor progression (29). A previous study has confirmed that Sox4 as an oncogene was overexpressed in EC cancer (30). Moreover, Zhou et al. reported that propofol suppressed esophageal squamous cell carcinoma cell migration and invasion by down-regulation of Sox4 (31). Based on these previous studies, we explored the relationship between propofol and Sox4 in EC cells. We found that propofol remarkably down-regulated Sox4 expression in a dose-dependent manner. In addition, propofol together with Sox4 overexpression significantly increased the proportion of Ki67^+^ cells. These data indicated that Sox4 overexpression might alleviate the anti-proliferative effect of propofol on EC cells. Our results from the *in vivo* study showed that propofol significantly inhibited tumor weight. However, the inhibitory effect of propofol on tumor formation was abolished by overexpression of Sox4. Overall, these data indicated that Sox4 might be a key regulator in EC.

Wnt/β-catenin signal pathway plays an important role in cell proliferation and tumor procession (32,33). In terms of EC, Han et al. reported that high mobility group protein A1 (HMGA1) modulated EC cell migration and invasion by activating Wnt/β-catenin signal pathway (34). Wang et al. found that progesterone blocked Wnt/β-catenin signal pathway in EC (35). However, the relationship of propofol, Sox4, and Wnt/β-catenin signal pathway in EC remain unclear. In our study, we demonstrated that propofol remarkably inhibited nuclear β-catenin expression in a dose-dependent and time-dependent manner. Moreover, Sox4 expression was significantly up-regulated by β-catenin overexpression but down-regulated by β-catenin silencing. Our study indicated that propofol inhibited Sox4 expression via inactivation of Wnt/β-catenin signal pathway.

In conclusion, our study demonstrated that propofol inhibited cell proliferation, migration, and invasion but promoted apoptosis by regulation of Sox4 in EC cells. Our results might provide a new insight for the treatment of EC. Further studies to explore the wider role of propofol in EC are still needed.
